# Genetically Determined Phenotype Covariation Networks Control Bone Strength

**DOI:** 10.1002/jbmr.41

**Published:** 2010-01-29

**Authors:** Karl J Jepsen, Hayden-William Courtland, Joseph H Nadeau

**Affiliations:** 1 Leni and Peter W May Department of Orthopaedics, Mount Sinai School of MedicineNew York, NY, USA; 2 Department of Genetics, Case Western Reserve University School of MedicineCleveland, OH, USA

**Keywords:** systems genetics, recombinant inbred mouse strains, bone, morphology, biomechanics, growth, phenotypic covariation, QTL, strength, chromosome substitution strains

## Abstract

To identify genes affecting bone strength, we studied how genetic variants regulate components of a phenotypic covariation network that was previously shown to accurately characterize the compensatory trait interactions involved in functional adaptation during growth. Quantitative trait loci (QTLs) regulating femoral robustness, morphologic compensation, and mineralization (tissue quality) were mapped at three ages during growth using AXB/BXA Recombinant Inbred (RI) mouse strains and adult B6-i^A^ Chromosome Substitution Strains (CSS). QTLs for robustness were identified on chromosomes 8, 12, 18, and 19 and confirmed at all three ages, indicating that genetic variants established robustness postnatally without further modification. A QTL for morphologic compensation, which was measured as the relationship between cortical area and body weight, was identified on chromosome 8. This QTL limited the amount of bone formed during growth and thus acted as a setpoint for diaphyseal bone mass. Additional QTLs were identified from the CSS analysis. QTLs for robustness and morphologic compensation regulated bone structure independently (ie, in a nonpleiotropic manner), indicating that each trait may be targeted separately to individualize treatments aiming to improve strength. Multiple regression analyses showed that variation in morphologic compensation and tissue quality, not bone size, determined femoral strength relative to body weight. Thus an individual inheriting slender bones will not necessarily inherit weak bones unless the individual also inherits a gene that impairs compensation. This systems genetic analysis showed that genetically determined phenotype covariation networks control bone strength, suggesting that incorporating functional adaptation into genetic analyses will advance our understanding of the genetic basis of bone strength. © 2010 American Society for Bone and Mineral Research.

## Introduction

A major challenge with studying the genetic basis of fracture susceptibility is that bone strength and clinically useful surrogate measures of strength such as bone mineral density (BMD) depend on multiple genetically defined structural and tissue-quality traits that change over time. This complexity is problematic for genetic analyses because it means that individuals within a population are at risk of fracture for different genetic reasons at different times and after different life histories. Most genetic analyses have used BMD or single physical bone traits to identify genetic variants contributing to fracture susceptibility.(1–4) However, this approach does not consider the complex, adaptive (ie, homeostatic) mechanisms that match bone stiffness and strength with physiologic loads during growth(5) and with aging.(6) These adaptive processes involve the simultaneous coordination of multiple traits, making it difficult to relate strength to any single physical bone trait. Considering the contribution of these adaptive mechanisms to the genetic regulation of strength is important because recent work has shown that robust bones and slender bones are functionally adapted in different ways during growth so that individuals with a wide range of external bone sizes can achieve similar strengths relative to body size.(7–11)

The functional adaptation process that establishes bone stiffness and strength during growth involved coordinated changes among morphologic and compositional traits.(10) We identified a pattern to this coordination that was consistent with engineering principles and that may be useful in genetic analyses. This pattern was not fully discernible from bivariate correlations but was better represented as a phenotypic covariation network.(7) The pattern of compensatory relationships among intermediate traits that were observed in the mouse skeleton(7,10,11) also was observed in the human skeleton(8,9) despite genetic and metabolic differences. This phenotypic covariation network may complicate the use of single traits to detect quantitative trait loci (QTLs) regulating bone strength because it means that variation in strength may not be predictable from a single trait but will depend on how multiple traits compensate for one another. Thus, incorporating functional adaptation into genetic analyses may benefit efforts to identify QTLs that act outside mechanisms that would not be discernible through variation in a single trait (eg, BMD, section modulus, etc.).

Biomechanical mechanisms leading to fracture susceptibility can be identified using a systems genetics approach, which uses genetic variants to study interactions among traits within biologic systems and determines how these interactions establish and maintain organ- or system-level function.(12) This approach has led to the discovery of new mechanisms leading to disease susceptibility and has provided new insight into the functionality of complex physiologic systems.(13–18) This systems genetics approach shifts the focus from understanding how genetic factors regulate bone size, shape, and mass to understanding how genetic factors regulate the processes that are involved in functional adaptation. Functional adaptation determines adult bone strength and thus is important to study for understanding fracture susceptibility later in life.

**Fig. 1:**
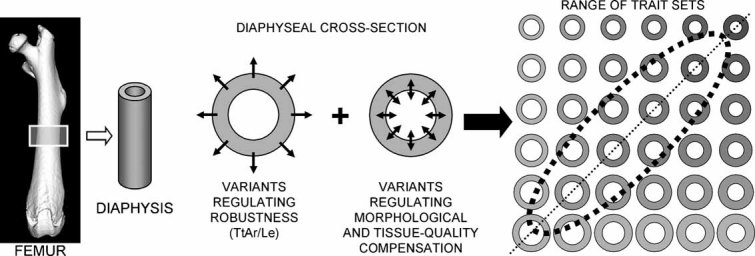
Schematic showing how sets of mid-diaphyseal traits acquired by an individual arise from variation in subperiosteal expansion defining robustness combined with variation in marrow infilling defining diaphyseal bone mass (cortical area) and mineralization defining tissue quality. These growth processes are highly coordinated, resulting in a population showing a narrow range of trait sets that is predictable based on bone robustness. The dashed diagonal line indicates the trait sets in which stiffness is maximized using minimum mass for a population. The gray value represents the variation in mineralization that accompanies morphologic compensation. The dashed elliptical line indicates the expected range of trait sets for a population showing variation in morphologic compensation.

We and others studied the homeostatic interactions among traits by determining how the human and mouse skeletal systems adapt to the natural variation in growth patterns that give rise to tremendous heterogeneity in bone size.(7–11) Variants affecting robustness, a measure of cross-sectional size relative to length, generally are tolerated in the mouse and human skeletons because compensatory changes in morphology and tissue quality strengthen slender phenotypes (narrow relative to length) and minimize mass in robust phenotypes (wide relative to length). The variation in robustness and the associated phenotypic covariation network resulted in individuals acquiring a set of functionally adapted traits that supported physiologic loads (Fig. 1). An emergent property of the phenotypic covariation network was that a population showed a narrow range of trait sets that was predictable based on robustness.(8,19) Because the phenotypic covariation network was important at a biomechanical level, we postulated that it also would be important at a genetic level.

Functionally or developmentally integrated sets of traits are generally thought to be regulated by a common gene (pleiotropy), a small set of genes (modularity), or nongenetically by a common signal imposed during development.(20–22) Pleiotropy and epistasis play important roles in skeletal trait variation and trait covariation in the context of anatomic function.(23) Pleiotropy among measures of bone strength and bone morphology also has been reported.(24,25) However, very little is known about the genetic architecture that regulates the process of functional adaptation that defines how well a bone is adapted to support physiologic loads. Solving this problem is important because variation in functional adaptation will affect adult bone strength and fracture risk. Presently, it is unclear whether the traits within the phenotypic covariation network that give rise to a functionally adapted bone also will be regulated in a pleiotropic manner.

To identify genetic variants affecting bone strength, we studied how genetic variants regulate specific aspects of the phenotypic covariation network that defines mechanical function.(7) We tested whether the growth processes specifying robustness and those specifying mechanical compensation are regulated by the same genes. Determining whether genetic variants regulate these growth processes independently is important not only for identifying biomechanical mechanisms linking genetic variants with bone strength but also for developing novel treatments that target a specific biologic process or that evoke compensatory changes in other traits to increase strength. The goals of this study were to (1) map QTLs regulating bone robustness and phenotypic covariation, (2) determine if these QTLs act independently, and (3) determine how these genetic factors affect the development of bone strength.

Mapping QTLs that regulate the interactions among traits has its challenges because genetic variants could affect each trait separately or the relationship between them.(26) We approached this problem from the perspective that a better understanding of how individual traits and the relationship among traits evolve during growth would help to map QTLs and would provide clues to the biologic processes regulated by the genetic variants. Mapping QTLs at multiple time points can be facilitated with inbred mouse strains rather than segregating crosses. We used a panel of AXB/BXA Recombinant Inbred (RI) mouse strains to map QTLs for traits measured at specific ages during growth. We further tested for genetic alterations in trait covariation using a panel of adult B6-Chr^A/J^/NaJ chromosome substitution strains (CSS).(27) The CSS panel was constructed by substituting chromosomes, one at a time, from the A/J strain (slender phenotype) onto the genetic background of the C57BL/6J (B6) strain (robust phenotype). QTLs are easily identified by directly comparing traits in CSS mice with the B6 host strain using straightforward statistical analyses.(28) Recent work examining adult CSS mice identified chromosomal substitutions that altered skeletal traits as well as body weight, body fat, total-body BMD, and serum insulin-like growth factor 1 (IGF-1).(29,30) However, these studies did not examine the compensatory interactions among traits that define bone strength.(7,10,11)

## Methods

### Husbandry

Female A/J, C57BL/6J (B6), and 20 AXB/BXA RI mouse strains (*n* = 8 to 17/age/strain) were obtained from the Jackson Laboratory (Bar Harbor, ME, USA). Individual cohorts were examined at 1 day and 4, 8, and 16 weeks of age, as described previously.(10) A panel of female B6-Chr^A/J^/NaJ CSSs were obtained from the original breeding colonies at Case Western Reserve University (Cleveland, OH, USA).(27,31) In some cases, mice were obtained from the Jackson Laboratory. Female CSS mice were euthanized at 16 weeks for phenotypic analysis (*n* = 58 for B6 mice, *n* = 6 to 12/strain for the CSS mice). Reciprocal F_1_ crosses were generated by intercrossing female and male A/J and B6 mice purchased from the Jackson Laboratory. Female AB6F_1_ (*n* = 10) and B6AF_1_ (*n* = 10) were euthanized at 15 weeks of age. Mice were fed a standard rodent diet (Purina Rodent Chow 5001; Purina Mills, Richmond, IN, USA) and water *ad libitum*, subjected to a 12-hour light/dark cycle, and raised with no more than 5 mice per cage. A total of 939 mice were examined in this study. The handling and treatment of mice were approved by the Institutional Animal Care and Use Committee.

### Bone robustness

Robustness has been measured in various ways,(32) with most formulas normalizing a measure of cross-sectional size (width, area, moment of inertia) by bone length (Le). We defined robustness as Tt.Ar/Le (see below) to be consistent with the fact that cross-sectional size increases proportional to the square of bone width (ie, area) during growth. Femoral length (Le) was measured from the proximal femoral head to the distal condyles using digital calipers (0.01-mm resolution). Cross-sectional morphologic traits, which included total area (Tt.Ar), cortical area (Ct.Ar), and marrow area (Ma.Ar), were measured at the femoral midshaft immediately distal to the third trochanter using standard histologic procedures (1-day-, 4-week-, and 8-week-old RI mice; all CSS and F_1_ mice) or from 3D images acquired using an eXplore Locus SP Pre-Clinical Specimen Micro-Computed Tomography System (GE Healthcare, London, Ontario, Canada; 16-week-old RI mice). Morphologic traits were quantified for each cross section in the analysis region, and the values were averaged. Trait values were reported previously for the RI(10) and CSS(29) panels. Tt.Ar/Le correlated weakly with body weight for the RI panel (not shown) and thus was not corrected for body size. However, body weight varied widely among the CSS panel, and consequently, regression analysis was used to correct Tt.Ar/Le for body weight for each CSS.

Path analysis(33) was conducted to determine whether variation in robustness among the RI panel arises because of variation in growth in length or variation in growth in width when the effects of body weight were taken into consideration (LISREL, Version 8.8; Scientific Software International, Lincoln Park, IL, USA). Body weight can be a confounding variable for skeletal traits because bigger mice tend to have bigger bones. Trait values were *Z*-transformed for each age group separately, as described previously.(10) Body weight, femur length, and Tt.Ar were treated as independent variables, and Tt.Ar/Le was treated as the dependent variable. Because only direct relationships were specified among the traits, this path analysis was equivalent to a multiple regression analysis. However, the relationships among traits were viewed in graphic format and in terms of standardized regression coefficients to show the relative contribution of each trait to robustness. Despite the differences in units for body weight, bone length, and total area, all traits varied linearly relative to one another during growth (data not shown).

### Morphologic compensation

Prior work showed that variation in bone size covaried with cortical thickness in a way that resulted in all RI strains using similar amounts of bone (Ct.Ar) to build functional structures during growth.(10) This relationship also was observed for the human femoral neck.(8) When similar amounts of tissue are packed into different spaces, wide (robust) bones will show reduced percent cortical area, whereas narrow (slender) bones will show increased percent cortical area. Thus, fixing the amount of tissue relative to body size is the mathematical basis for morphologic compensation in long bones. For femoral diaphyses, the “amount of tissue” is measured as cortical area (Ct.Ar). Because cortical area increases linearly with body weight across growth,(10) the Ct.Ar:BW ratio provides a simple measure of the amount of morphologic compensation. This ratio was calculated for each sample in the RI panel at the 4-, 8-, and 16-week time groups using a linear regression–based method. Cortical area for each sample was calculated as


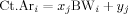
(1)

where *i* is the sample number, *j* refers to the RI strain, and *x*_*j*_ and *y*_*j*_ are the slope and *y* intercept respectively, calculated for each RI strain by regressing Ct.Ar and BW using data collected at 4, 8, and 16 weeks. Normalizing for body weight gives


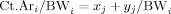
(2)

A Ct.Ar/BW ratio was calculated for each sample, and the values were averaged for each RI strain. The slope *x*_*j*_ and the Ct.Ar/BW ratio measured at each age were used in the QTL analysis. Because data for the CSS panel and the reciprocal F_1_ crosses were obtained at 15 to 16 weeks only, Ct.Ar/BW was measured by calculating this ratio directly without using the regression-based method. A regression analysis comparing Ct.Ar/BW measured using 16-week data for the RI panel versus data collected throughout growth (Eq. 2) showed a significant linear regression (*r*^2^ = 0.99, *p* < .0001) between the two measures, indicating that calculating the Ct.Ar–BW relationship using adult data alone provided a reasonable approximation of this relationship.

### Degree of mineralization

In addition to morphology, the quality of the bone tissue also contributes to whole-bone function. Tissue quality can encompass a variety of physical traits, including the organic and inorganic components of the mineral and matrix,(34) as well as the tissue-level organization of these factors.(35) In this investigation, we quantified tissue quality in terms of the degree of mineralization because mineral content correlates positively with tissue stiffness and strength but negatively with tissue ductility.(34,36) For the RI samples, micro-computed tomography (µCT) was used to quantify tissue mineral density (TMDn) for femurs at 16 weeks of age, as described previously.(7) TMDn (mg/cc) was determined by converting grayscale values to mineral density values using a density calibration factor and then averaging mineral content values over all thresholded “bone” voxels. The density calibration factor was determined for each scan using a phantom containing air, water, and a hydroxyapatite standard (SB3; Gammex RMI, Middleton, WI, USA). TMDn determined by µCT for over 20 inbred mouse strains correlated linearly with traditional ash-content measures (*p* < .001; unpublished data). For the CSSs and reciprocal F_1_ crosses, mineralization was measured with standard methods.(37) Ash-content analyses were performed on the femoral diaphyses after mechanical testing. Diaphyses were cleaned of extraneous soft tissue and the marrow expelled. Hydrated, dry, and ash weights were determined as described previously.(37) Ash content was calculated for each sample as the percentage of ash weight relative to hydrated weight.

### QTL analysis

Linkage analysis was conducted using the mean values for each of the 20 RI strains to map QTLs regulating bone robustness, the Ct.Ar/BW ratio, and the degree of mineralization. The strain distribution patterns (SDPs) for the RI strains were downloaded from the Mouse Genome Informatics Project (http://www.informatics.jax.org). The SDPs for the AXB/BXA RI panel include 811 markers across 19 autosomes and the X chromosome. Closely spaced (redundant) marker loci (<10 cM) were removed, leaving a total of 530 markers for linkage analysis. QTLs were detected by marker regression and localized by interval mapping using Map Manager QTX software (Version 0.30, Roswell Park Cancer Institute, Buffalo, NY, USA).(38) Interval mapping was conducted by fitting a regression equation for the effect of a hypothetical QTL at the position of each marker locus and at regular intervals between marker loci.(39) Confidence intervals (CIs) were estimated by bootstrap resampling.(40) A likelihood-ratio statistic (LRS) was generated as a measure of significance for QTL detection (LRS = 4.6 × LOD score). Suggestive (37th percentile), significant (95th percentile), and highly significant (99.9th percentile) LRS scores were determined on a genome-wide basis by permutation testing (1000 times).(41) Because several RI strains (AXB18, AXB19, and AXB20) were not considered independent owing to greater than 86% similarity in strain distribution patterns,(42) marker regression analyses were run with AXB18 removed to confirm the significance levels of the QTLs. AXB19 was retained for marker regression analysis because Tt.Ar/Le and Ct.Ar/BW differed from one or both AXB18 and AXB19 at each age (*p* < .02, ANOVA). For the CSSs, chromosomes harboring QTLs regulating bone traits were identified by comparing trait values of each CSS (19 autosomes, X) with the B6 host using a *t* test. The threshold *p* value for individual *t* tests (*p* < .004) was corrected for multiple comparisons to achieve a genome-wide significance level of *p* < .05.(28)

### Independence of QTL effects

To determine if the QTLs for robustness and Ct.Ar/BW regulate skeletal function independently, trait values for the RI strains with different combinations of B6 and A/J alleles for robustness and Ct.Ar/BW were compared at 16 weeks of age using ANOVA. In addition, the number of CSSs showing significant changes in robustness alone, Ct.Ar/BW alone, and ash content alone were counted, along with the number of CSSs showing changes in any combination of these traits.

### Effect of genetic factors on skeletal function

To determine how the genetically regulated traits such as robustness, Ct.Ar/BW ratio, and degree of mineralization affect the development of bone strength, left femurs from 16-week-old B6, A/J, RI, and CSS mice were subjected to destructive testing to assess whole-bone mechanical properties. Femurs were loaded to failure in four-point bending at 0.05 mm/s using a servohydraulic materials testing system (Instron Corp, Canton, MA, USA) following established protocols.(37) Load-deflection curves were analyzed for several mechanical properties, including stiffness (the slope of the initial portion of the curve) and maximum load. Means and SDs for each property were measured for each inbred mouse strain.

Because bone stiffness and maximum load generally increase with body size, each mechanical property was regressed against body weight, and the residuals were calculated as a measure of how well the femurs of each inbred strain was functionally adapted to support body weight. A positive residual indicated that a bone was overdesigned (stronger) relative to body size, whereas a negative residual indicated that a bone was underdesigned (weaker) relative to body size. Because different technologies were used to measure mineral content, the analysis was conducted using *Z*-transformed data to simplify comparison of the RI and CSS regression equations. A multivariate linear regression analysis was conducted to determine how the QTLs regulating robustness and compensation affected mechanical function.

## Results

### Mode of inheritance

**Fig. 2:**
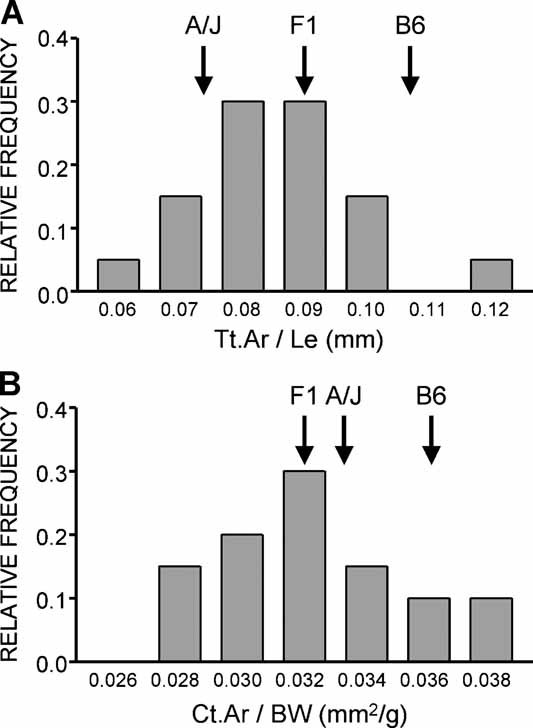
Variation in (*A*) robustness (Tt.Ar/Le) and (*B*) morphologic compensation (Ct.Ar/BW) among the AXB/BXA RI panel. Mean trait values are shown for female A/J, B6, and F_1_ inbred strains. Trait values for AB6F1 and B6AF1 strains were similar and are indicated as an average F_1_.

Trait values of the RI panel and the reciprocal F_1_ intercrosses were compared with the progenitor strains to assess mode of inheritance. Femurs from AB6F1 and B6AF1 strains were not different for any morphologic trait (*p* > .2, *t* test). Likewise, no differences in the average Tt.Ar/Le or Ct.Ar/BW values were found between the AXB and BXA RI strains (*p* > .3, *t* test). However, B6AF1 femurs showed a 3.8% greater mineralization (*p* < .01, *t* test) compared with AB6F1 femurs. This paternal inheritance mode for mineralization (A/J > B6) also was observed in the AXB/BXA RI strains such that BXA RI strains showed a 1.9% greater mineralization than AXB RI strains (*p* < .07, *t* test). Additional RI strains will have to be analyzed to determine whether the paternal inheritance pattern observed for the reciprocal F_1_ cross remains significant following multiple generations of intercrosses in the RI panel. The average Tt.Ar/Le values at 16 weeks for AB6F1 (0.087 ± 0.006 mm), B6AF1 (0.090 ± 0.004 mm), and RI (0.085 ± 0.013 mm) femurs were intermediate between A/J and B6 (Fig. 2*A*). In contrast, the Ct.Ar/BW values at 16 weeks for AB6F1 (0.032 ± 0.001 mm^2^/g), B6AF1 (0.032 ± 0.002 mm^2^/g), and RI (0.032 ± 0.003 mm^2^/g) femurs were lower than those in both progenitor strains (Fig. 2*B*), indicating a modest overdominant inheritance mode.

### QTLs for robustness

**Fig. 3:**
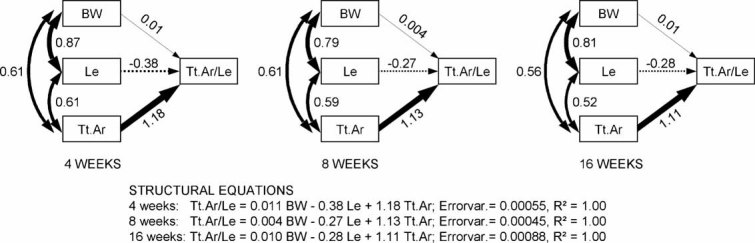
Path analysis was conducted to determine how variation in Tt.Ar and Le among the RI strains contributed to the variation in robustness (Tt.Ar/Le) when the effects of body weight were taken into consideration. The coefficients next to the curved arrows in the path models are the linear correlations between independent variables, and the coefficients next to the straight arrows are the relative contributions of each trait to Tt.Ar/Le in terms of standardized units (*Z*-scores). A large path coefficient was observed for Tt.Ar at each age, indicating that most of the variation in robustness was determined by Tt.Ar. Length contributed to the variation in Tt.Ar/Le but to a much lesser extent. The relative contributions were determined after controlling for the effects of body weight, which correlated significantly with Tt.Ar and Le but contributed very little to the variation in Tt.Ar/Le. The analyses were consistent across growth, suggesting that variation in robustness among the RI strains resulted primarily from variation in growth in width.

Marker regression analysis was conducted using the RI panel to map QTLs regulating robustness. All AXB/BXA RI traits were normally distributed (Kolmogorv-Smirnov test, *p* > .1). The threshold level for suggestive QTLs was LRS = 10.2 (LOD = 2.2), and this value was similar for all traits examined. Because the 95% significance level varied among the traits, significant LRS values were determined for traits individually. The LRS significance level (*p* < .05) ranged from 14.3 (LOD = 3.1) to 19.8 (LOD = 4.3) with an average of 17.1 (LOD = 3.7). Four chromosomes harboring suggestive and significant QTLs were identified for Tt.Ar/Le, and these were replicated at 4, 8, and 16 weeks of age (Table 1). The similarity in QTLs identified at the three ages suggested that the genetic factors that define the relationship between growth in width and growth in length were established early and sustained across growth. A similar set of QTLs was identified for Tt.Ar but not Le (Table 2). This can be explained by the path analysis showing that most of the variation in robustness at each age was determined by Tt.Ar (Fig. 3). RI strains inheriting the B6 allele on chromosomes 8, 12, and 18 had significantly greater Tt.Ar/Le values at all ages compared with the RI strains inheriting the A/J allele (eg, 8 weeks of age: Tt.Ar/Le = 0.101 ± 0.009 mm for the B6 allele versus 0.082 ± 0.008 for the A/J allele; *p* < .0001, *t* test). However, the opposite was true for chromosome 19; RI strains with the A/J allele had significantly greater robustness than those with the B6 allele (eg, 8 weeks of age: Tt.Ar/Le = 0.083 ± 0.008 mm for the B6 allele versus 0.104 ± 0.009 for A/J allele; *p* < .0001, *t* test).

**Table 1:** QTLs for Robustness (Tt.Ar/Le) at Three Ages

			4 weeks			8 weeks			16 weeks		
					
Chromosome	Position (cM)	Marker	LRS	% Variation	CI	LRS	% Variation	CI	LRS	% Variation	CI
8	37.0	D8Mit305	12.6 **↑**	47	—	**17.4 ↑**	58	36	14.2 **↑**	51	—
12	6.0	D12Mit58	**16.9 ↑**	57	38	**17.5 ↑**	58	36	**15.4 ↑**	54	48
18	20.0	D18Mit17	**15.3 ↑**	54	48	**16.6 ↑**	56	40	12.7 **↑**	47	—
19	20.0	D19Mit86	11.3 **↓**	43	—	**16.9 ↓**	57	39	12.8 **↓**	47	—

*Note:* Significant QTLs are indicated in boldface. Significance levels for Tt.Ar/Le: LRS = 15.2 at 4 weeks, 16.4 at 8 weeks, and 14.9 at 16 weeks. % Variance is the percentage of total trait variance explained by the QTL at each locus. CI is the width (in cM) of the 95% confidence interval and is reported only for significant QTLs. Arrows indicate direction of effect for the B6 alleles.

**Table 2:** QTLs for Total Cross-Sectional Area (Tt.Ar) and Femoral Length (Le) at Three Ages

				4 weeks			8 weeks			16 weeks		
						
Chromosome	Trait	Position (cM)	Marker	LRS	% Variation	CI	LRS	% Variation	CI	LRS	% Variation	CI
7	Le	38.0	D7J5	**18.5 ↓**	**60**	32	n.s.	—	—	n.s.	—	—
8	Tt.Ar	37.0	D8Mit305	n.s.	—	—	16.1 **↑**	55	—	12.0 **↑**	45	—
12	Tt.Ar	6.0	D12Mit58	16.2 **↑**	56	—	**18.3 ↑**	60	33	16.1 **↑**	55	—
18	Tt.Ar	20.0	D18Mit17	16.1 **↑**	55	—	**18.7 ↑**	61	31	13.0 **↑**	48	—

*Note:* Significant QTLs are indicated in boldface. Significance levels for Tt.Ar: LRS = 16.9 at 4 weeks, 18.2 at 8 weeks, and 17.2 at 16 weeks. Significance levels for Le: LRS = 17.3 at 4 weeks. % Variation is the percentage of total trait variance explained by the QTL at each locus. CI is the width (in cM) of the 95% confidence interval and is reported only for significant QTLs. Arrows indicate direction of effect for the B6 alleles.

**Fig. 4:**
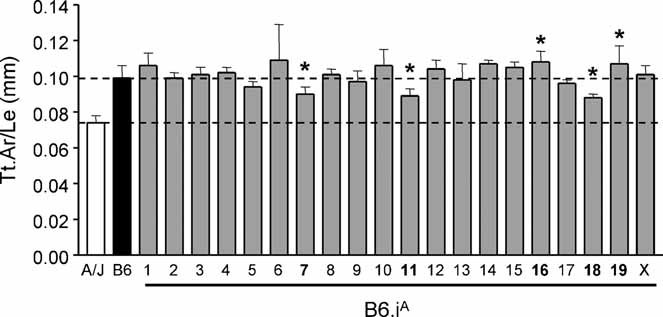
Variation in femoral robustness (Tt.Ar/Le) at 16 weeks of age for the panel of female CSSs. Tt.Ar/Le was corrected for body weight by linear regression analysis. Data are shown as mean ± SD. The asterisk indicates significant differences between each CSS and the B6 host (*p* < .004, *t* test).

A comparison of trait values between each CSS and the B6 host strain identified five chromosome substitutions that resulted in significantly more slender (CSS-7, CSS-11, and CSS-18) or more robust (CSS-16 and CSS-19) phenotypes (Fig. 4). A significant difference between a CSS and the B6 host strain indicates that the chromosome harbors one or more QTLs regulating robustness. This analysis confirmed that the QTLs mapped to chromosomes 18 and 19 but not 8 and 12 from the analysis of the RI strains. Analysis of the CSS panel also confirmed that the A/J allele on chromosome 18 negatively affected robustness, whereas the A/J allele on chromosome 19 positively affected robustness.

### QTLs for covariation

**Fig. 5:**
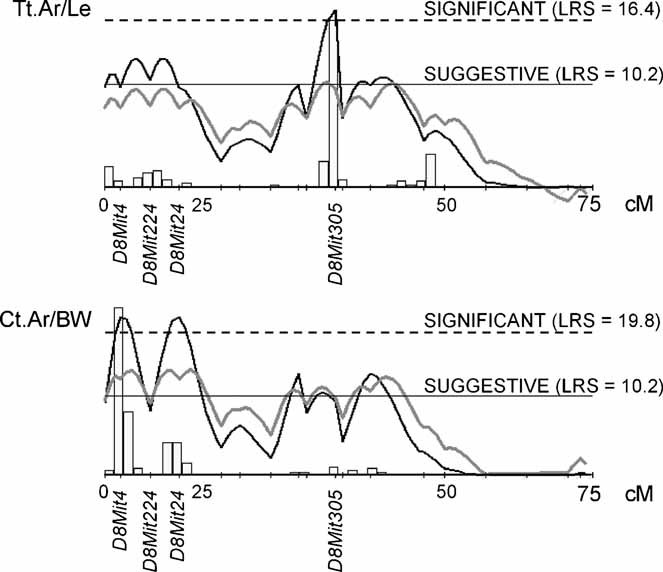
Interval mapping showed that QTLs for robustness (Tt.Ar/Le) and morphologic compensation (Ct.Ar/BW) measured at 8 weeks of age localized to different regions on chromosome 8. The solid black line represents the LRS level and the solid gray line represents the additive effect. The horizontal solid and dashed black lines represent the suggestive and significant (95th percentile) LRS levels, respectively. The vertical bars are the histograms showing the range of the maximum LRS values.

For analysis of the RI strains, no significant or suggestive QTLs were identified for TMDn or the slope of the Ct.Ar–BW regressions calculated using data collected at 4, 8, and 16 weeks of age. However, a significant QTL was mapped to chromosome 8 for the Ct.Ar/BW ratio calculated at 8 weeks of age (Table 3). Interval mapping showed that the QTLs identified on chromosome 8 for Tt.Ar/Le and Ct.Ar/BW localized to different chromosomal regions (Fig. 5), indicating that these traits were regulated by distinct loci. RI strains inheriting the B6 allele on chromosome 8 (D8Mit4 or D8Mit24) had a greater Ct.Ar/BW ratio than the RI strains inheriting the A/J allele (0.034 ± 0.002 for the B6 allele versus 0.031 ± 0.003 for the A/J allele, *p* < .03). A borderline significant QTL was identified on chromosome 11 (D11Mit38) for Ct.Ar/BW measured at 16 weeks. A suggestive QTL at this same marker also was identified at 4 weeks. Suggestive QTLs were identified on chromosomes 5 (4 and 8 weeks) and 16 (4 weeks).

**Table 3:** QTLs for Ct.Ar/BW Measured at Three Ages

			4 weeks			8 weeks			16 weeks		
					
Chromosome	Position (cM)	Marker	LRS	% Variation	CI	LRS	% Variation	CI	LRS	% Variation	CI
5	28	D5Mit55	n.s.	—	—	17.0 **↑**	57	—	n.s.	—	—
8	14–18	D8Mit4/24	n.s.	—	—	**21.9 ↑**	67	25	n.s.	—	—
11	49	D11Mit38	12.0 ↑	45	—	n.s.	—	—	17.7 **↑**	59	—
16	27.3	D16Mit4	14.1 ↓	51	—	n.s.	—	—	n.s.	—	—

*Note:* Significant QTLs are indicated in boldface. Significance levels for Ct.Ar/BW: LRS = 18.9 at 4 weeks, 19.8 at 8 weeks, and 18.1 at 16 weeks. % Variation is the percentage of total trait variance explained by the QTL at each locus. CI is the width (in cM) of the 95% confidence interval and is reported only for significant QTLs. Arrows indicate direction of effect for B6 alleles.

**Fig. 6:**
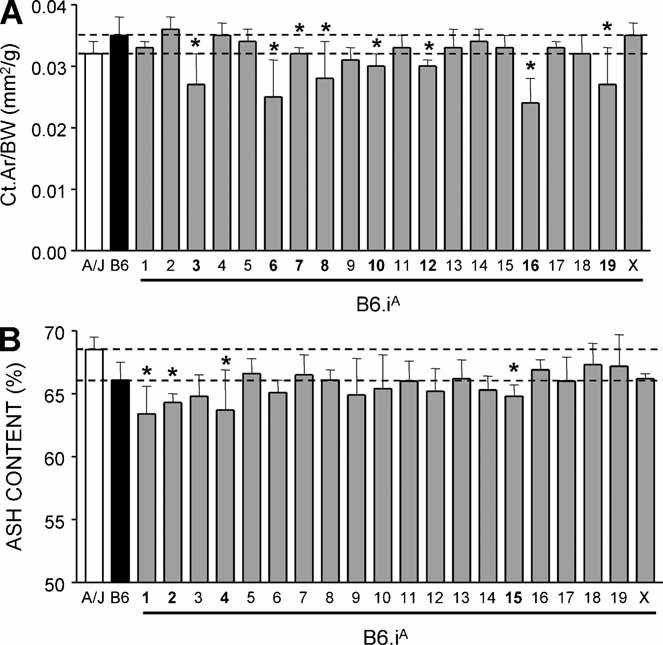
Variation in (*A*) morphologic compensation (Ct.Ar/BW) and (*B*) ash content at 16 weeks of age for the panel of female CSSs. Data are shown as mean ± SD. The asterisk indicates significant differences between each CSS and the B6 host (*p* < .004, *t* test).

Analysis of the CSS panel identified eight chromosome substitutions that significantly reduced Ct.Ar/BW values compared with the B6 host strain (CSS-3, CSS-6, CSS-7, CSS-8, CSS-10, CSS-12, CSS-16, and CSS-19; Fig. 6*A*). This analysis confirmed the significant covariation QTL on chromosome 8 and the suggestive covariation QTL on chromosome 16 identified in the RI analysis. Further, four CSSs showed reduced ash content compared with the B6 host (CSS-1, CSS-2, CSS-4, and CSS-15), indicating that QTLs regulating mineralization reside on these chromosomes (Fig. 6*B*).

### Combined effects of QTLs regulating robustness and Ct.Ar/BW on adult bone structure

**Fig. 7:**
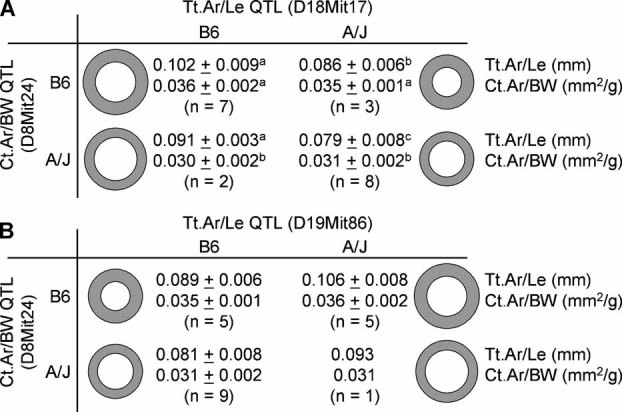
Effect of B6 and A/J genotypes on the trait sets inherited by the AXB/BXA RI strains. The femoral diaphyseal morphologic traits (mean ± SD) were measured at 8 weeks of age. (*A*) The effect of genotype for Ct.Ar/BW on chromosome 8 and for Tt.Ar/Le on chromosome 18 on diaphyseal morphology. The letters (*a*, *b*, *c*) indicate differences among groups based on a two-way ANOVA. (*B*) The effect of genotype for Ct.Ar/BW on chromosome 8 and for Tt.Ar/Le on chromosome 19 on diaphyseal morphology.

Trait values for the RI strains with different combinations of B6 and A/J alleles for robustness and Ct.Ar/BW were compared at 16 weeks of age to determine how these variants together affected bone structure. RI strains inheriting the B6 allele on chromosome 18 for robustness tended to have larger Tt.Ar/Le values, and those inheriting the A/J allele had lower Tt.Ar/Le values regardless of what allele was inherited for Ct.Ar/BW (*p* < .0004, ANOVA; Fig. 7*A*). Likewise, RI strains inheriting the B6 allele for Ct.Ar/BW had larger values for Ct.Ar/BW, and those inheriting the A/J allele for Ct.Ar/BW had lower Ct.Ar/BW values (*p* < .0003, ANOVA) regardless of what allele was inherited for robustness. Thus RI strains with a B6-B6 genotype for robustness and Ct.Ar/BW, respectively, acquired robust bones with large Ct.Ar/BW values, RI strains with a B6-A/J genotype acquired robust bones with small Ct.Ar/BW values, RI strains with an A/J-B6 genotype acquired slender bones with large Ct.Ar/BW values, and RI strains with an A/J-A/J genotype acquired slender bones with small Ct.Ar/BW values. Similar results were observed for chromosomes 8, 12, and 19, but the effects of the B6 and A/J alleles on robustness were reversed for chromosome 19 (Fig. 7*B*). Thus the QTLs regulating robustness and Ct.Ar/BW appeared to exert independent effects on bone traits in the RI panel. Likewise, analysis of the CSS panel identified two chromosome substitutions that significantly altered Tt.Ar/Le alone (CSS-11 and CSS-18) and five substitutions that significantly altered Ct.Ar/BW alone (CSS-3, CSS-6, CSS-8, CSS-10, and CSS-12). However, three substitutions significantly altered both traits (CSS-7, CSS-16, and CSS-19). CSS-7 had slender bones with reduced Ct.Ar/BW values, whereas CSS-16 and CSS-19 had robust bones with reduced Ct.Ar/BW values. These chromosomes may harbor multiple QTLs having individual effects, or this may be evidence that the same QTL exerts effects on both traits. None of the CSSs showing significantly reduced ash content overlapped with the CSSs showing significantly altered morphology.

### Variation in mechanical function is determined by genetic factors regulating compensation

A multivariate linear regression analysis was conducted to determine how the QTLs regulating robustness and compensation affected mechanical function. The multivariate regression analysis of the RI data showed that 58.8% (*p* < .002) of the stiffness–body weight residuals and 61.5% (*p* < .001) of the maximum load–body weight residuals were explained by mineral content, Tt.Ar/Le, and Ct.Ar/BW measured at 16 weeks of age (Table 4). Ct.Ar/BW was the only significant predictor of the stiffness–body weight and maximum load–body weight residuals, indicating that the amount of morphologic compensation was a significant determinant of whether an RI strain acquired traits leading to an overdesigned or underdesigned femur during growth. Likewise for the CSS panel, 46.2% (*p* < .017) of the stiffness–body weight residuals and 51.4% (*p* < .008) of the maximum load–body weight residuals were determined by mineral content, Tt.Ar/Le, and Ct.Ar/BW measured at 16 weeks of age. Unlike the analysis of the RI strains, mineral content was the significant predictor of the stiffness–body weight residuals, but both mineral content and Ct.Ar/BW were significant predictors of the maximum load–body weight residuals. The multivariate analysis indicated that mechanical function depended on compensation, not size.

**Table 4:** Multivariate Linear Regression Analysis

Strain	Equation	*r*^2^
RI	Stiff-BW Res = 0.32 mineral – 0.12 Tt.Ar/Le + 0.88 **Ct.Ar/BW**	58.8
RI	Max-BW Res = 0.19 mineral + 0.09 Tt.Ar/Le + 0.78 **Ct.Ar/BW**	61.5
CSS	Stiff-BW Res = 0.67 **mineral** – 0.04 Tt.Ar/Le + 0.38 Ct.Ar/BW	46.2
CSS	Max-BW Res = 0.74 **mineral** + 0.33 Tt.Ar/Le + 0.75 **Ct.Ar/BW**	51.4

*Note:* Traits with individual *p* < .02 are shown in boldface. Stiff-BW Res = stiffness–body weight residuals; Max-BW Res = maximum load–body weight residuals

## Discussion

### Altered phenotypic covariation links QTLs with bone strength

Establishing mechanical homeostasis of long bones during growth involves compensatory interactions among certain morphologic and tissue-quality traits that are superimposed on genetic variation in robustness.(7,10) Using mouse B6-i^A^ CSS and AXB/BXA RI strains, we mapped several QTLs regulating Ct.Ar/BW, a measure of the amount of morphologic compensation, as well as mineral content, a measure of tissue quality. The QTL analysis showed that trait covariation in the context of functional adaptation has an important genetic basis. The multiple regression analysis showed that variations in Ct.Ar/BW and mineral content were the primary determinants of whether femurs were weak or strong relative to body size. This analysis indicated that bone strength depended on the QTLs regulating morphologic compensation and mineralization, but not simply bone size. Finding that QTLs regulate the interactions among traits that define mechanical function helped to explain, in biomechanical terms, how genetic variants are linked to bone strength and fragility.

Although interindividual variation in external bone size has been documented for the human skeleton,(43) the compensatory changes that accompany this variation in external size have not been incorporated into genetic studies. QTLs for bone size, composition, and strength have been reported in mouse models,(24,25,44–48) but these studies did not test whether QTLs regulating single traits were associated with compensatory changes in other traits that act to stabilize whole-bone stiffness and strength. Although changes in bone size are important for strength, our analysis showed that genetic variants affect bone strength in a more complex way. This complexity can be attributed to variation in the phenotypic covariation network that was shown to capture the functional adaptation process of the mouse skeletal system.(7) Variation in compensation would have the effect of expanding the width of the ellipse in Fig. 1, which characterizes the range of trait sets acquired by individuals within a population. Our data suggest that mice inheriting slender (or robust) bones do not necessarily inherit weak bones, unless they also inherit a gene that impairs morphologic or tissue-quality compensation. We expect that there is a minimum robustness value that can be compensated given biologic and biomechanical constraints defining the minimum amount of marrow infilling and the maximum degree of matrix mineralization. The range of robustness and the limitations on compensation have not been fully established.

The idea that genetic variants affecting compensation rather than size are responsible for alterations in system function (ie, fitness) is not new. Further, the genetic regulation of functional interactions among traits is not limited to inbred mouse strains but also has been observed in outbred populations.(25,45,46,49–51) It is generally acknowledged that the integration of morphologic traits helps to buffer the skeletal system from certain variants (eg, variation in mandibular length) and that fitness depends on how variants affect the coordination among traits (eg, variation in mandibular length has little effect on food processing if accompanied by properly coordinated changes in maxillary length).(22) Morphologic integration has been studied extensively in many species primarily in the context of anatomic function, evolutionary changes in skeletal shape, pleiotropy, and modular genetic architecture.(20–22,26,52,53) Cheverud(22) differentiated among different levels of morphologic integration. At the individual level, the relationships among morphologic traits involve developmental and functional integration, which refers to traits that interact during development or are directed by a common stimulus (eg, mechanical force or growth hormone). At the population level, which is the primary focus of our study, functional and developmental integration at the individual level results in genetic integration across a population.(22) Genetic integration refers to the inheritance of functional sets of morphologic traits. Our data showed that integration is not limited to anatomic function but also applies to mechanical function. Compensatory interactions within the phenotypic covariation network resulted in the inheritance of sets of morphologic and compositional traits that were predictable across a population based on bone robustness. Because the phenotypic covariation network of AXB/BXA RI mouse strains was similar to that of the human skeleton,(7–11) using the mouse to study how QTLs regulate components of the phenotypic covariation network may lead to important new approaches for studying the genetic regulation of strength in the human skeleton. Further work is needed to determine whether the similarities between the mouse and human skeletons are limited to covariation among intermediate traits or if these systems also share similar genetic controls of trait covariation.

### Genetic factors regulating robustness

Mapping QTLs regulating robustness is important not only because they document naturally occurring genetic variants that are generally tolerated under daily physiologic conditions but also because slenderness is a risk factor for fracture under extreme load conditions in the elderly,(54,55) children,(56) military recruits,(57) and young-adult athletes.(58) The QTLs we identified for robustness in this study were replicated at multiple time points using independent cohorts of RI mice, suggesting that the genetic factors that define robustness were expressed early during growth and were not modified significantly by other factors during and after puberty. This is consistent with prior work showing that robustness at 4 weeks of age is highly correlated with robustness measured at 16 weeks in RI mice.(10) Likewise, in the human skeleton, robustness is established early during postnatal growth (approximately 2 years of age) and stabilized thereafter despite continued overall growth.(19,59)

QTLs for femoral cross-sectional size have been mapped in the mouse to within 6 cM of the QTLs identified for the AXB/BXA RI strains on chromosome 8,(60,61) 12,(60) and 18.(62) Analysis of the CSSs identified additional QTLs for robustness, which have been mapped to chromosomes 7,(60,63,64) 11,(46,61,65) and 16(46) in other studies. The QTL on chromosome 19 identified using the RI strains appears to be novel. The genetic and functional analyses across growth provided important clues to the expected biologic function of the genes regulating robustness. The robustness QTLs affected femurs by altering transverse expansion but not longitudinal growth, by sustaining the relationship between growth in width and growth in length through adulthood, and by not altering the compensatory changes in morphology or tissue quality.

### Genetic factors regulating compensation

Compensation for variation in robustness can occur through changes in morphology as well as tissue quality. The multiple regression analysis showed that compensation via mineralization was critically important for establishing whole-bone stiffness and strength for the CSS panel. Tissue quality contributes substantially to overall stiffness and strength and is particularly important for slender structures.(10) Mineral content has not been widely incorporated into genetic analyses. QTLs for mineral content have been mapped to chromosomes 7, 9, 12, and 15 in other work.(46) QTLs for tissue hardness, which correlates with matrix mineralization,(66) were mapped to chromosomes 8, 12, 13, 17, and 19.(67) The QTLs on chromosomes 1, 2, and 4 identified by our analysis of the CSS appear to be novel. No QTLs for mineral content were mapped using the RI data, and this may be attributed in part to alterations in the strain distribution pattern resulting from the paternal inheritance pattern of ash content.

In addition to tissue quality, we also examined Ct.Ar/BW, which serves the dual role of defining the degree of morphologic compensation and establishing a setpoint for bone mass, similar to that described by Frost in his mechanostat theory.(68) It was unclear from our data whether this setpoint is defined by metabolic, neurologic, or mechanical factors. Unlike total cross-sectional area, which is established early during growth, cortical area is determined over the entire growth period and involves cellular events (ie, infilling and expansion) on the endocortical surface. Because of cortical drift, growth of diaphyseal size and cross-sectional area results from the actions of both osteoblasts and osteoclasts. Morphologic compensation may be less important for slender structures because adding bone to the endocortical surface has limited benefit to overall stiffness.(10) However, morphologic compensation is a critical factor for minimizing mass in robust structures to avoid acquiring excessive bulk that is metabolically expensive to maintain. The QTLs identified using the RI strains mapped to different chromosomal regions for the three ages examined. This was expected because prior work showed that the slopes and intercepts of the Ct.Ar–BW regressions varied among the RI strains.(10) The variable slopes and intercepts changed the distribution of Ct.Ar/BW values across the RI panel at each age, and thus linkage to QTLs varied with each age examined. The variable slopes and intercepts may indicate that the biologic processes that establish the Ct.Ar–BW relationship vary during growth. This QTL colocalized with a femur breaking strength phenotype(25) and a mechanosensitivity phenotype(69) identified by others. The suggestive QTL for Ct.Ar/BW on chromosome 11 colocalized with a QTL for serum Insulin-like Growth Factor Binding Protein-5 (IGFBP-5).(70) Although the Ct.Ar/BW ratio has not been studied previously, QTLs for body weight–corrected cortical area (females) have been mapped to chromosomes 1,(46) 4,(62) 5,(46) and 6.(62) QTLs for Ct.Ar also were identified in 18-month-old mice on chromosomes 3, 4, 8, and 15, but these were not corrected for body weight.(61)

Because the QTL linked to Ct.Ar/BW acts like a setpoint for diaphyseal bone mass, the data suggested that a gene in this QTL region functions like a limit switch regulating the amount of bone that can be used to construct a femur during growth. This gene poses a particularly important problem for fragility in the adult skeleton because the intent is to build the appropriate amount of bone relative to external size during growth so that the adult structure is sufficiently stiff and strong to support physiologic loads without being excessively bulky. A gene that limits diaphyseal bone mass (ie, cortical area) despite functional loading demands will significantly threaten overall adult strength and increase fracture susceptibility with age-related bone loss. Whether this QTL also alters bone mass in corticocancellous structures has yet to be determined. Although slenderness has been identified as being an important risk factor for fractures,(54–58) investigating the relationship between robustness and Ct.Ar/BW may help to explain another important structural feature that also has been associated with fracture risk in human studies. Work by others showed that having wide bones combined with proportionally thin cortices is a risk factor for fractures in the elderly.(71,72) How adults acquire this particular set of traits is not fully understood. Although bone structure and tissue quality are modified during aging, variation in sets of adult traits was overwhelmingly attributable to growth effects and surprisingly little to aging effects.(8) Variable skeletal growth patterns are largely responsible for generating the sex- and race-specific trait sets(73,74) that contribute to varying fracture rates among populations.(75) Our data using mouse models indicated that genetic background regulates the amount of morphologic compensation during growth and thus defines the particular set of traits acquired as an adult and then taken through the aging process. Consequently, studying the genetic basis of how bone regulates compensation is highly relevant and of great importance.

### Components of the phenotypic covariation network are regulated independently

There has been tremendous progress in identifying traits that are coregulated by a common gene (pleiotropy).(22,76) As noted by Cheverud, genetic coregulation of traits necessarily results in phenotypic correlation.(22) However, the reverse is not always true: Phenotypically correlated traits do not necessarily result from pleiotropy.(50) Several studies in mice and humans showed that QTLs regulating morphologic traits and mechanical properties tend to map to similar chromosomal regions.(25,45,46,50) This has been attributed generally to pleiotropy or linked loci. Multivariate analyses such as principal-component analysis also identified pleiotropic QTLs(44) but did not reveal the compensatory trait interactions required to establish mechanical function. We showed that for a simple structure such as the femoral diaphysis, multiple traits are coordinated in a systematic way to establish function.(7,10) This coordination increased in complexity for a corticocancellous structure such as the vertebral body.(11)

Prior work from our laboratory showed that patterns of trait sets across a population result from the integration of traits (functional adaptation) during development.(10) The current data do not support the idea that the functional interactions among these traits result from either pleiotropy or linkage disequilibrium. In fact, the current data argue strongly against this idea. On dissecting the components of the phenotypic covariation network, we found that despite functional interactions among traits within this network, the underlying trait controlling QTLs acted largely independently and thus in a nonpleiotropic manner. Analysis of the RI data showed that robustness and Ct.Ar/BW, which are critical components of the phenotypic covariation network, were regulated independently. Analysis of the CSS data showed that most of the chromosome substitutions affected robustness, Ct.Ar/BW, and mineralization independently and that only 3 of 14 chromosome substitutions showed effects on more than one system component. Because many genes reside on each chromosome, further work is needed to determine whether the phenotypes for the three chromosome substitutions (B6.7A, B6.16A, and B6.19A) showing effects on more than one system component arose from a pleiotropic QTL or multiple independent loci residing on these chromosomes. This can be answered through the generation of congenic lines and the analysis of phenotypes across growth. Our analysis of mouse femoral diaphyses suggested that external size and cortical area are regulated independently, and thus each process may be targeted independently to individualize prophylactic treatments that aim to increase bone strength. The data do not, however, reveal whether the QTLs affected a common biologic process that was responsible for the coordination of these traits. This important question will have to be studied in future work.

The lack of a pleiotropic effect in the RI panel may be a consequence of the limited power of this technology to detect only QTLs with major effects.(77) Further, the confidence intervals, which were determined by bootstrap resampling, depend on the effect of the QTL and the population size.(40) Additional work is needed to refine the location of the QTLs within the confidence intervals. The CSS panel is a more sensitive technology for detecting QTLs.(27) However, the CSS data were limited to whole-chromosome substitutions, and these may or may not be able to confirm the RI results depending on the complexity of the genetic regulatory elements within each chromosome.(17) Future work will use congenics derived from this panel to further understand the genetic architecture regulating compensation and to confirm the QTLs identified from the RI analysis. The lack of pleiotropy in our analysis also could result from the variable times during growth in which the functional interactions arise. This time dependence suggests that the coordination of traits may involve different biologic processes. Mineralization and robustness are integrated early postnatally because variants affecting subperiosteal expansion rate are inversely related to the degree of matrix mineralization by 2 to 4 weeks of age in mice.(10,78) The coordination between subperiosteal expansion rate and the relative cortical area (Ct.Ar/Tt.Ar) occurs throughout growth but appears to mature during puberty. Genetic variants can impair the development of a functionally adapted set of adult traits depending on the timing in which a mutation affects growth. For example, liver insulin-like growth factor 1 (IGF-1) deficiency leads to a slender adult phenotype but does not elicit the expected compensatory increase in mineral compensation because subperiosteal expansion was impaired well after postnatal growth.(79) This mutation lead to significantly reduced adult bone strength. However, work by others provided examples of how certain matrix mutations(80) and exercise(81) were associated with compensatory changes in tissue quality that appeared well after the postnatal growth phase. These studies emphasize that much work needs to be done to better understand the complexity of trait compensation and how variation in compensation affects strength.

## Conclusion

Combining engineering principles with genetic analyses advanced our understanding of the genetic basis of fragility by identifying genetic regulation of phenotypic covariation networks as an important biomechanical mechanism linking genetic variants with bone strength. The genetic variants linked to robustness and morphologic compensation affected bone independently, indicating that each of these traits may be targeted separately to improve bone strength by individualizing prophylactic treatments.

## References

[1] Liu YJ, Shen H, Xiao P (2006). Molecular genetic studies of gene identification for osteoporosis: a 2004 update. J Bone Miner Res..

[2] Ralston SH (2007). Genetics of osteoporosis. Proc Nutr Soc..

[3] Xiong Q, Han C, Beamer WG, Gu W (2008). A close examination of genes within quantitative trait loci of bone mineral density in whole mouse genome. Crit Rev Eukaryot Gene Expr..

[4] Chen Y, Shen H, Yang F (2009). Choice of study phenotype in osteoporosis genetic research. J Bone Miner Metab..

[5] Ruff C, Holt B, Trinkaus E (2006). Who's afraid of the big bad Wolff? “Wolff's law” and bone functional adaptation. Am J Phys Anthropol..

[6] Smith RW, Walker RR (1964). Femoral expansion in aging women: Implications for osteoporosis and fractures. Science.

[7] Jepsen KJ, Hu B, Tommasini SM (2007). Genetic randomization reveals functional relationships among morphologic and tissue-quality traits that contribute to bone strength and fragility. Mamm Genome.

[8] Zebaze RM, Jones A, Knackstedt M, Maalouf G, Seeman E (2007). Construction of the femoral neck during growth determines its strength in old age. J Bone Miner Res.

[9] Tommasini SM, Nasser P, Hu B, Jepsen KJ (2008). Biological Co-adaptation of Morphological and Composition Traits Contributes to Mechanical Functionality and Skeletal Fragility. J Bone Miner Res.

[10] Jepsen KJ, Hu B, Tommasini SM (2009). Phenotypic integration of skeletal traits during growth buffers genetic variants affecting the slenderness of femora in inbred mouse strains. Mamm Genome.

[11] Tommasini SM, Hu B, Nadeau JH, Jepsen KJ (2009). Phenotypic integration among trabecular and cortical bone traits establishes mechanical functionality of inbred mouse vertebrae. J Bone Miner Res.

[12] Churchill GA (2007). Recombinant inbred strain panels: a tool for systems genetics. Physiol Genomics.

[13] Rutherford SL (2000). From genotype to phenotype: buffering mechanisms and the storage of genetic information. Bioessays.

[14] Rutherford SL, Lindquist S (1998). Hsp90 as a capacitor for morphological evolution. Nature.

[15] Marder E, Goaillard JM (2006). Variability, compensation and homeostasis in neuron and network function. Nat Rev Neurosci.

[16] Llamas B, Belanger S, Picard S, Deschepper CF (2007). Cardiac mass and cardiomyocyte size are governed by different genetic loci on either autosomes or chromosome Y in recombinant inbred mice. Physiol Genomics.

[17] Millward CA, Burrage LC, Shao H (2009). Genetic factors for resistance to diet-induced obesity and associated metabolic traits on mouse chromosome 17. Mamm Genome.

[18] Yingling VR (2009). A delay in pubertal onset affects the covariation of body weight, estradiol, and bone size. Calcif Tissue Int.

[19] Pandey N, Bhola S, Goldstone A (2009). Inter-individual variation in functionally adapted trait sets is established during post-natal growth and predictable based on bone robusticity. J Bone Miner Res Epub..

[20] Olson EC, Miller RL (1958). Morphological Integration.

[21] Cheverud JM (1982). Phenotypic, genetic, and environmental morphological integration in the cranium. Evolution.

[22] Cheverud JM (1996). Developmental integration and the evolution of pleiotropy. American Zoologist.

[23] Wolf JB, Pomp D, Eisen EJ, Cheverud JM, Leamy LJ (2006). The contribution of epistatic pleiotropy to the genetic architecture of covariation among polygenic traits in mice. Evol Dev.

[24] Yershov Y, Baldini TH, Villagomez S (2001). Bone strength and related traits in HcB/Dem recombinant congenic mice. J Bone Miner Res.

[25] Li X, Masinde G, Gu W, Wergedal J, Mohan S, Baylink DJ (2002). Genetic dissection of femur breaking strength in a large population (MRL/MpJ x SJL/J) of F2 Mice: single QTL effects, epistasis, and pleiotropy. Genomics.

[26] Li R, Tsaih S-W, Shockley K (2006). Structural model analysis of multiple quantitative traits. PLoS Genetics.

[27] Nadeau JH, Singer JB, Matin A, Lander ES (2000). Analysing complex genetic traits with chromosome substitution strains. Nat Genet.

[28] Belknap JK (2003). Chromosome substitution strains: some quantitative considerations for genome scans and fine mapping. Mamm Genome.

[29] Shao H, Burrage L, Sinasac D (2008). Genetic architecture of complex traits: large phenotypic effects and pervasive epistasis. PNAS.

[30] Govoni KE, Donahue LR, Marden C, Mohan S (2008). Complex genetic regulation of bone mineral density and insulin-like growth factor-I in C57BL/6J-Chr #A/J/NaJ chromosome substitution strains. Physiol Genomics.

[31] Singer JB, Hill AE, Burrage LC (2004). Genetic dissection of complex traits with chromosome substitution strains of mice. Science.

[32] Pearson OM (2000). Activity, climate, and postcranial robusticity: implications for modern human origins and scenarios of adaptive change. Curr Anthropol.

[33] Grace JB (2006). Structural Equation Modeling and Natural Systems.

[34] Courtland H-W, Nasser P, Goldstone AB, Spevak L, Boskey AL, Jepsen KJ (2008). FTIRI microspectroscopy and micromechanical testing reveal intra-species variation in mouse bone mineral composition and matrix maturity. Calcif Tissue Int.

[35] Skedros JG, Dayton MR, Sybrowsky CL, Bloebaum RD, Bachus KN (2006). The influence of collagen fiber orientation and other histocompositional characteristics on the mechanical properties of equine cortical bone. J Exp Biol.

[36] Currey JD (1984). Effects of differences in mineralization on the mechanical properties of bone. Philos Trans R Soc Lond B Biol Sci.

[37] Jepsen KJ, Pennington DE, Lee YL, Warman M, Nadeau J (2001). Bone brittleness varies with genetic background in A/J and C57BL/6J inbred mice. J Bone Miner Res.

[38] Manly KF, Cudmore RH, Meer JM (2001). Map Manager QTX, cross-platform software for genetic mapping. Mamm Genome.

[39] Haley CS, Knott SA (1992). A simple regression method for mapping quantitative trait loci in line crosses using flanking markers. Heredity.

[40] Visscher PM, Thompson R, Haley CS (1996). Confidence intervals in QTL mapping by bootstrapping. Genetics.

[41] Churchill GA, Doerge RW (1994). Empirical threshold values for quantitative trait mapping. Genetics.

[42] Sampson SB, Higgins DC, Elliot RW (1998). An edited linkage map for the AXB and BXA recombinant inbred mouse strains. Mamm Genome.

[43] Garn S (1970). The earlier gain and the later loss of cortical bone.

[44] Koller DL, Schriefer J, Sun Q (2003). Genetic effects for femoral biomechanics, structure, and density in C57BL/6J and C3H/HeJ inbred mouse strains. J Bone Miner Res.

[45] Volkman SK, Galecki AT, Burke DT, Miller RA, Goldstein SA (2004). Quantitative trait loci that modulate femoral mechanical properties in a genetically heterogeneous mouse population. J Bone Miner Res.

[46] Lang DH, Sharkey NA, Mack HA (2005). Quantitative trait loci analysis of structural and material skeletal phenotypes in C57BL/6J and DBA/2 second-generation and recombinant inbred mice. J Bone Miner Res.

[47] Alam I, Sun Q, Liu L (2005). Whole-genome scan for linkage to bone strength and structure in inbred Fischer 344 and Lewis rats. J Bone Miner Res.

[48] Rubin CJ, Brandstrom H, Wright D (2007). Quantitative trait loci for BMD and bone strength in an intercross between domestic and wildtype chickens. J Bone Miner Res.

[49] Wolf JB, Leamy LJ, Routman EJ, Cheverud JM (2005). Epistatic pleiotropy and the genetic architecture of covariation within early and late-developing skull trait complexes in mice. Genetics.

[50] Karasik D, Dupuis J, Cupples LA (2007). Bivariate linkage study of proximal hip geometry and body size indices: the Framingham study. Calcif Tissue Int.

[51] Norgard EA, Roseman CC, Fawcett GL (2008). Identification of quantitative trait loci affecting murine long bone length in a two-generation intercross of LG/J and SM/J Mice. J Bone Miner Res.

[52] Klingenberg CP, Leamy LJ, Cheverud JM (2004). Integration and modularity of quantitative trait locus effects on geometric shape in the mouse mandible. Genetics.

[53] Klingenberg CP, Mebus K, Auffray JC (2003). Developmental integration in a complex morphological structure: how distinct are the modules in the mouse mandible?. Evol Dev.

[54] Albright F, Smith PH, Richardson AM (1941). Post-menopausal osteoporosis. Its clinical features. JAMA.

[55] Szulc P, Munoz F, Duboeuf F, Marchand F, Delmas PD (2006). Low width of tubular bones is associated with increased risk of fragility fracture in elderly men--the MINOS study. Bone.

[56] Landin L, Nilsson BE (1983). Bone mineral content in children with fractures. Clin Orthop Relat Res.

[57] Milgrom C, Giladi M, Simkin A (1989). The area moment of inertia of the tibia: a risk factor for stress fractures. J Biomech.

[58] Crossley K, Bennell KL, Wrigley T, Oakes BW (1999). Ground reaction forces, bone characteristics, and tibial stress fracture in male runners. Med Sci Sports Exerc.

[59] Bonnard G (1968). Cortical thickness and diaphysial diameter of the metacarpal bones from the age of three months to eleven years. Helv Paediatr Acta.

[60] Klein RF, Turner RJ, Skinner LD (2002). Mapping quantitative trait loci that influence femoral cross-sectional area in mice. J Bone Miner Res.

[61] Volkman SK, Galecki AT, Burke DT (2003). Quantitative trait loci for femoral size and shape in a genetically heterogeneous mouse population. J Bone Miner Res.

[62] Turner CH, Sun Q, Schriefer J (2003). Congenic mice reveal sex-specific genetic regulation of femoral structure and strength. Calcif Tissue Int.

[63] Drake TA, Hannani K, Kabo JM, Villa V, Krass K, Lusis AJ (2001). Genetic loci influencing natural variations in femoral bone morphometry in mice. J Orthop Res.

[64] Wergedal JE, Ackert-Bicknell CL, Tsaih SW (2006). Femur mechanical properties in the F2 progeny of an NZB/B1NJ x RF/J cross are regulated predominantly by genetic loci that regulate bone geometry. J Bone Miner Res.

[65] Masinde GL, Wergedal J, Davidson H (2003). Quantitative trait loci for periosteal circumference (PC): identification of single loci and epistatic effects in F2 MRL/SJL mice. Bone.

[66] Miller LM, Little W, Schirmer A, Sheik F, Busa B, Judex S (2007). Accretion of bone quantity and quality in the developing mouse skeleton. J Bone Miner Res.

[67] Jiao Y, Chiu H, Fan Z (2007). Quantitative trait loci that determine mouse tibial nanoindentation properties in an F2 population derived from C57BL/6J x C3H/HeJ. Calcif Tissue Int.

[68] Frost HM (1987). Bone “mass” and the “mechanostat”: a proposal. Anat Rec.

[69] Robling AG, Warden SJ, Shultz KL, Beamer WG, Turner CH (2007). Genetic effects on bone mechanotransduction in congenic mice harboring bone size and strength quantitative trait loci. J Bone Miner Res.

[70] Mohan S, Masinde G, Li X, Baylink DJ (2003). Mapping quantitative trait loci that influence serum insulin-like growth factor binding protein-5 levels in F2 mice (MRL/MpJ X SJL/J). Endocrinology.

[71] Gluer CC, Cummings SR, Pressman A (1994). Prediction of hip fractures from pelvic radiographs: the study of osteoporotic fractures. The Study of Osteoporotic Fractures Research Group. J Bone Miner Res.

[72] Kaptoge S, Beck TJ, Reeve J (2008). Prediction of incident hip fracture risk by femur geometry variables measured by hip structural analysis in the study of osteoporotic fractures. J Bone Miner Res.

[73] Duan Y, Beck TJ, Wang XF, Seeman E (2003). Structural and biomechanical basis of sexual dimorphism in femoral neck fragility has its origins in growth and aging. J Bone Miner Res.

[74] Wang XF, Duan Y, Beck TJ, Seeman E (2005). Varying contributions of growth and ageing to racial and sex differences in femoral neck structure and strength in old age. Bone.

[75] Cummings SR, Melton LJ (2002). Epidemiology and outcomes of osteoporotic fractures. Lancet.

[76] Christians JK, Senger LK (2007). Fine mapping dissects pleiotropic growth quantitative trait locus into linked loci. Mamm Genome.

[77] Belknap JK, Mitchell SR, O'Toole LA, Helms ML, Crabbe JC (1996). Type I and type II error rates for quantitative trait loci (QTL) mapping studies using recombinant inbred mouse strains. Behav Genet.

[78] Price CP, Herman BC, Lufkin T, Goldman HM, Jepsen KJ (2005). Genetic variation in bone growth patterns defines adult mouse bone fragility. J Bone Miner Res.

[79] Yakar S, Canalis E, Sun H (2009). Serum IGF-1 determines skeletal strength by regulating sub-periosteal expansion and compensatory trait interactions. J Bone Miner Res.

[80] Kozloff KM, Carden A, Bergwitz C (2004). Brittle IV mouse model for osteogenesis imperfecta IV demonstrates postpubertal adaptations to improve whole bone strength. J Bone Miner Res.

[81] Wallace JM, Ron MS, Kohn DH (2009). Short-term exercise in mice increases tibial post-yield mechanical properties while two weeks of latency following exercise increases tissue-level strength. Calcif Tissue Int.

